# Stachydrine from Natural Foods Alleviates Hyperuricemia by Modulating Renal Urate Transporters and Suppressing Mitochondrial Oxidative Stress

**DOI:** 10.3390/foods14101718

**Published:** 2025-05-12

**Authors:** Jian Guo, Jinhui Jia, Ailin Wang, Yunqi Gu, Xiaodong Xia

**Affiliations:** 1 State Key Laboratory of Marine Food Processing and Safety Control, National Engineering Research Center of Seafood, School of Food Science and Technology, Dalian Polytechnic University, Dalian 116034, China; guojianbio@sina.com (J.G.); jjh05271998@163.com (J.J.); wal0630@163.com (A.W.); 13470398760@163.com (Y.G.); 2 Dalian Jinshiwan Laboratory, Dalian 116034, China

**Keywords:** stachydrine, hyperuricemia, inflammation, mitochondrial damage, oxidative stress, Keap1/Nrf2

## Abstract

Hyperuricemia (HUA) is a metabolic disease caused by disrupted purine metabolism, characterized by abnormally elevated uric acid (UA) levels. Stachydrine, an alkaloid in natural foods, exhibits multiple biological activities. This study aimed to evaluate the effects of stachydrine on alleviating HUA. An HUA mouse model was established through high-nucleoside diet induction, and stachydrine’s effects on UA levels and renal injury were investigated. Our findings revealed that stachydrine enhanced uric acid excretion by upregulating ATP-binding cassette subfamily G member 2 (ABCG2). Furthermore, stachydrine mitigated HUA-induced renal inflammation, mitochondrial oxidative stress and apoptosis. Mechanistically, stachydrine facilitated the nuclear translocation of nuclear factor erythroid 2-related factor 2 (Nrf2) by downregulating Kelch-like ECH-associated protein 1 (Keap1), subsequently activating the Keap1/Nrf2 signaling pathway and alleviating local oxidative stress. This study demonstrated the UA-lowering and renoprotective effects of stachydrine, suggesting its potential as a functional food ingredient for mitigating HUA.

## 1. Introduction

Hyperuricemia (HUA) is a prevalent metabolic disease characterized by disrupted uric acid (UA) homeostasis. In humans, UA is the terminal product in purine metabolism, with hepatic synthesis followed by elimination via renal (70%) and enteric (30%) routes [1]. Pathological conditions leading to excessive synthesis or obstructed excretion result in the continued accumulation of UA, eventually causing HUA (UA ≥ 420 μM in males or ≥360 μM in females) [2].

HUA independently increases the risk of chronic kidney disease [3]. The kidney is the primary excretory organ for UA. High concentrations of UA activate the NLRP3/NF-κB signaling cascade, upregulating pro-inflammatory cytokines interleukin (IL)-18, IL-1β and tumor necrosis factor-α (TNF-α), thereby triggering renal inflammation [4]. HUA further promotes renal damage via oxidative stress pathways. As a potent antioxidant, UA has an antioxidant capacity comparable to that of vitamin C, but its level is 10-fold higher in the blood than vitamin C [5]. High levels of UA accumulation are speculated to be a specific evolutionary adaptation to protect neurons from reactive oxygen species (ROS). The pro-oxidative activity of UA mainly occurs in cells. Although the specific mechanism of its dual effects is unclear, HUA exerts systemic impacts through oxidative stress-mediated pathways, particularly on renal tissues. HUA-induced oxidative stress promotes DNA damage, inflammatory responses, and apoptotic signaling [6]. Mitochondria are the primary organelles affected by cellular oxidative stress. Excessive ROS can disrupt the normal structure of mitochondria, leading to ATP depletion, ADP accumulation, and mitochondrial membrane potential (MMP) depolarization [7,8]. Chronic HUA exposure can induce mitochondrial dysfunction in rats, resulting in epithelial cell apoptosis and tubular damage [9]. The caspase-dependent mitochondrial cascade is a central mechanism of apoptosis. Mitochondrial damage promotes cytochrome C release, which binds to apoptotic protease-activating factor-1 (Apaf-1) and activates caspase-9, subsequently cleaving caspase-3 to execute apoptosis [10]. To counteract oxidative stress, the body has an endogenous defense system, such as superoxide dismutase (SOD) and glutathione (GSH), which scavenge excess ROS to maintain redox homeostasis. In addition, nuclear factor erythroid 2-related factor 2 (Nrf2) and Kelch-like ECH-associated protein 1 (Keap1) plays pivotal roles in suppressing oxidative stress. Activation of the Keap1/Nrf2 pathway upregulates a series of antioxidant enzymes, including glutamate cysteine ligase catalytic subunit (GCLC), NADPH-quinone oxidoreductase-1 (NQO1) and heme oxygenase-1 (HO-1) to synergistically resist oxidative stress [11].

Currently, effective strategies for treating HUA include inhibiting hepatic UA biosynthesis and modulating renal/intestinal urate transport systems. UA biosynthesis primarily involves xanthine oxidase (XOD) and adenosine deaminase (ADA), while XOD is the most clinically validated pharmacological target for HUA management [12,13]. UA homeostasis is further modulated by three key transporters: human urate transporter 1 (URAT1), which mediates renal UA reabsorption; glucose transporter 9 (GLUT9), involved in basolateral UA uptake; and ATP-binding cassette subfamily G member 2 (ABCG2), which facilitates UA secretion in both kidneys and intestines [14]. Impaired or insufficient UA excretion mediated by transporters induces HUA, promoting inflammatory responses and mitochondrial oxidative stress, which ultimately results in renal damage. As essential molecular targets, urate transporters provide complementary strategies for regulating systemic UA balance. Current clinical treatments for HUA primarily include allopurinol and febuxostat (both inhibitors of XOD), and benzbromarone, a URAT1 inhibitor that decreases UA reabsorption. However, potential adverse reactions to these drugs may include allergic manifestations, hepatotoxicity and gastrointestinal reactions [15,16].

**Figure 1:**
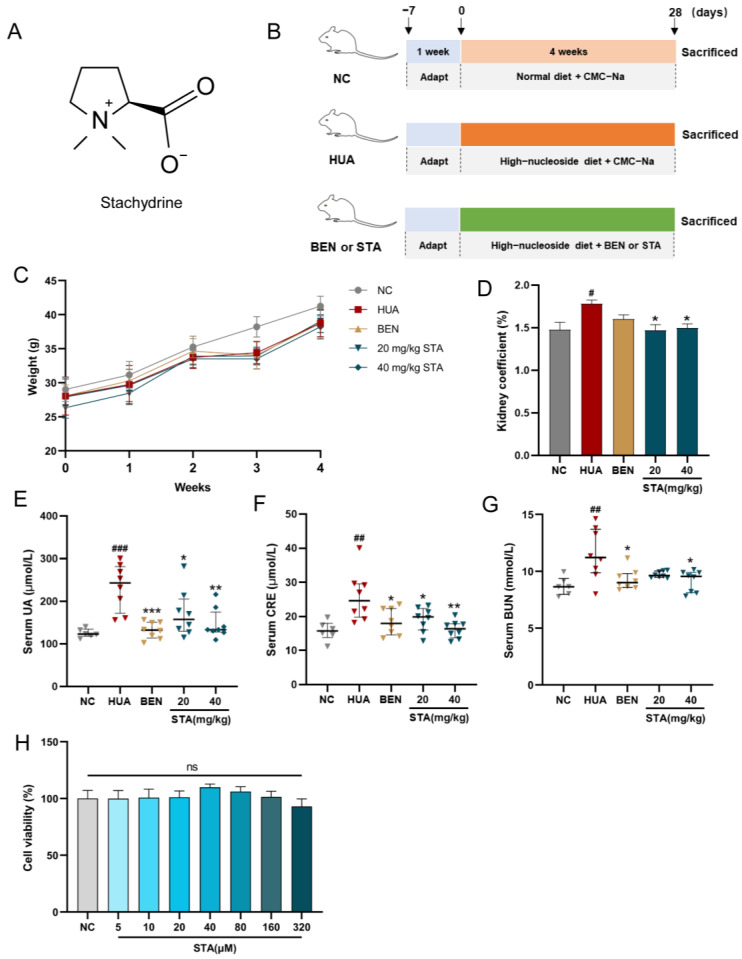
STA alleviated HUA in mice. (**A**) Chemical structure of STA. (**B**) Animal experiment design. (**C**) Body weight. (**D**) Kidney coefficient. (**E**–**G**) Serum UA, CRE and BUN. (**H**) Effects of STA on HK-2 cell viability. ^#^ *p* < 0.05, ^##^ *p* < 0.01, ^###^ *p* < 0.001 vs. NC group; * *p* < 0.05, ** *p* < 0.01, *** *p* < 0.001 vs. HUA group.

Stachydrine (STA), chemically designated as L-proline betaine, is an alkaloid commonly found in edible and medicinal plants, such as citrus (orange and grapefruit), chestnut, alfalfa and motherwort [17]. Its molecular structure is (2S)-1,1-dimethylpyrrolidine-2-carboxylic acid [18] (Figure 1A). STA exhibits various physiological functions, including the inhibition of myocardial cell fibrosis and the improvement of cardiac power and blood flow by inhibiting the TGF-β/Smad signaling pathway [19]. Moreover, STA demonstrates neuroprotective properties by attenuating oxidative stress and suppressing inflammatory responses, thus mitigating brain and nerve tissue injuries resulting from ischemia and hypoxia [20]. Studies have indicated that STA exhibits nephroprotective properties. In a rat model featuring unilateral ureteral obstruction, STA suppresses apoptosis induced by endoplasmic reticulum stress and attenuates renal interstitial fibrosis [21]. However, STA’s impact on HUA remains unexplored.

Interfering with UA metabolism by supplementing purine precursors could recapitulate the HUA process caused by excessive diet or purine metabolism disorder observed clinically. In this study, we established an HUA mouse model. A high-nucleoside diet containing excess inosine and guanosine and potassium oxonate (PO, a uricase inhibitor) was administered to induce sustained HUA phenotypes and renal damage. We hypothesized that STA could alleviate HUA by modulating urate transporters, and evaluated their renal expression levels. Mitochondrial dysfunction and cell apoptosis were assessed to evaluate STA’s impact on oxidative stress. The antioxidant mechanisms of STA were further explored in HK-2 renal epithelial cells.

## 2. Materials and Methods

### 2.1. Materials and Reagents

Stachydrine, potassium oxonate, inosine, guanosine and ML385 were obtained from Macklin (Shanghai, China). Assay kits for detecting UA, CRE, BUN, XOD, ADA, MDA, SOD, GSH and ATP were obtained from Nanjing Jiancheng (Nanjing, China). ELISA kits for IL-1β, IL-18, IL-10, TNF-α and ADP were acquired from Mlbio (Shanghai, China). Activity assay kits for caspase-3 and caspase-9, ROS assay kit, MMP assay kit (JC-1) and HRP-labeled goat anti-rabbit IgG (H + L) (A0208) were purchased from Beyotime (Shanghai, China). Antibodies targeting Nrf2 (A11159), Keap1 (A25951), β-actin (AC038) and Histone H3 (A2348) were obtained from ABclonal (Wuhan, China).

### 2.2. Animal Experiment

Six-week-old ICR mice (male) were purchased from Liaoning Changsheng Biotechnology Co., Ltd. (Shenyang, China). All experimental mice were housed in a specific pathogen-free environment with controlled temperature (22–26 °C) and humidity (40–60%). Food and drinking water were provided without restriction. After one week of adaptive feeding, mice were randomly assigned to experimental groups (*n* = 8/group). The animal experiment design is shown in Figure 1B. All reagents were suspended in a 0.5% sodium carboxymethyl cellulose (CMC-Na) aqueous solution. A high-nucleoside formula was prepared, containing 400 mg/kg potassium oxonate, 750 mg/kg inosine and 750 mg/kg guanosine. All interventions were performed by intragastric (IG) administration to ensure dosing consistency among individuals. Starting from Day 0, the HUA group received the high-nucleoside formula and vehicle (0.5% CMC-Na); the STA and benzbromarone (BEN) groups received the high-nucleoside formula, and were simultaneously treated with STA (20 or 40 mg/kg) or BEN (10 mg/kg), respectively; and the normal control (NC) group was given an equivalent volume of vehicle. During this period, all mice were fed a standard diet. After 4 weeks of treatment, mice were sacrificed under deep isoflurane anesthesia by cervical dislocation, and serum samples were collected. Kidney tissues were fixed with 4% paraformaldehyde or 2.5% glutaraldehyde, or rapidly stored at −80 °C. Feces and 24-h urine were collected the day before euthanasia.

### 2.3. Biochemical and Inflammatory Marker Analysis

The commercial assay kits were used to quantify the following indicators, including UA in serum, urine and feces; creatinine (CRE), blood urea nitrogen (BUN) in serum; XOD, ADA activity in serum; malondialdehyde (MDA), SOD, GSH, caspase-3, caspase-9, ATP, ADP and inflammatory markers in kidney tissues or HK-2 cells. ADP and inflammatory markers (IL-1β, IL-18, IL-10, TNF-α) were assayed using enzyme-linked immunosorbent assay (ELISA) kits. Detection was performed as per the manufacturer’s guideline.

### 2.4. RT-qPCR

Total RNA was isolated from kidney tissues and HK-2 cells with Trizole reagent (Sangon, Shanghai, China, B511311). Subsequently, cDNA synthesis was carried out using an Evo M-MLV RT Kit (AGBio, Hunan, China, AG11711). The RT-qPCR was conducted with a TB Green^®^ Premix Ex Taq™ II (Tli RNaseH Plus) (Takara, Kusatsu, Japan, RR820A), and the specific primer sequences are provided in Table 1.

### 2.5. Histopathological and Immunofluorescence Analysis

In hematoxylin and eosin (H&E) staining, following fixation with 4% paraformaldehyde, kidney tissue sections were processed through hematoxylin staining followed by eosin counterstaining. Renal pathological damage was observed, including alterations in glomerular morphology, tubular dilation and inflammatory infiltration. The kidney injury score was evaluated as previously described [22]. For terminal deoxynucleotidyl transferase dUTP nick-end labeling (TUNEL) staining, the sections were catalyzed by terminal deoxynucleotidyl transferase (TdT) and a fluorescent probe Cyanine 3 (Cy3) was added to evaluate cell apoptosis. For immunohistochemical staining, non-specific binding sites were incubated with 3% bovine serum albumin (BSA) for 30 min. Primary antibody incubation was performed on the sections (4 °C, overnight). Subsequently, HRP-labeled secondary antibodies were applied for 1 h at room temperature. 3,3′-Diaminobenzidine (DAB) and hematoxylin were subsequently utilized for the final staining. For immunofluorescence staining, incubation conditions for blocking and primary antibodies were the same as above. After that, fluorescently labeled secondary antibodies were incubated for 1 h. Nuclear staining was performed using 4′,6-Diamidino-2-phenylindole (DAPI). The stained results were observed using a light or fluorescence microscope.

### 2.6. Tissue DHE Analysis

Dihydroethidium (DHE) was used to detect ROS (superoxide anion) levels in kidney tissues. Specifically, 5 μM DHE was incubated with tissue sections (37 °C, 30 min), and ROS levels were evaluated by detecting the oxidation product visualized as red fluorescence.

### 2.7. Cell Culture

HK-2 cells were cultured in MEM (10% fetal bovine serum, FBS) under 5% CO_2_ at 37 °C, and seeded in culture plates at a density of 1 × 10^5^ cells/mL. The cells were exposed to STA (20 or 40 μM) with or without UA (200 μg/mL) for 36 h in 6-well plates, and then harvested for further analysis, including quantification of mRNA levels (GCLC, NQO1, HO-1) by RT-qPCR, detection of protein levels (Nrf2, Keap1, β-actin and Histone H3) by Western blot and immunofluorescence. The MMP, caspase-3 and caspase-9 activities in HK-2 cells were also detected to evaluate mitochondrial damage and apoptosis. In the Nrf2 pathway inhibition experiment, 5 μM ML385 was added during cell culture. Subsequently, the levels of ROS, MDA, SOD, GSH and the mRNA expression of GCLC, NQO1, HO-1 were measured. For viability assessment, cells in a 96-well plate were treated with different concentrations of STA for 48 h, and then the cell-counting kit-8 (CCK-8) reagent was added. Following 40 min incubation, cell viability was assessed by measuring absorbance at 450 nm.

### 2.8. Assessment of MMP and Cellular ROS

HK-2 cells were cultured and treated according to previous description. 2′,7′-Dichlorodihydrofluorescein diacetate (DCFH-DA) as a fluorescent probe was used to detect intracellular ROS levels. MMP was assessed using 5,5′,6,6′-Tetrachloro-1,1′,3,3′-tetraethylbenzimidazolylcarbocyanine iodide(JC-1) following the manufacturer’s protocol, with subsequent visualization by fluorescence microscopy.

### 2.9. Transmission Electron Microscope (TEM)

Kidney tissues fixed with 2.5% glutaraldehyde were sectioned into 80 nm slices, and then stained with uranyl acetate and lead citrate. Mitochondrial ultrastructure was observed with transmission electron microscope (HT7700, Hitachi, Tokyo, Japan).

### 2.10. Western Blot

Total protein was isolated from HK-2 cells using a nuclear/cytoplasmic protein ex-traction kit. Protein samples were resolved on 8% SDS-polyacrylamide gels and transferred onto polyvinylidene fluoride (PVDF) membranes. The membranes were immersed in 5% skim milk to block 1 h, followed by overnight incubation (4 °C) with antibodies targeting Nrf2, Keap1, β-actin and Histone H3. After washing, the blots were probed with HRP-labeled secondary antibody for 1 h. Protein bands were detected by the enhanced chemiluminescence (ECL) method. Band intensities were quantified with ImageJ software (Fiji, v1.54f; NIH, Bethesda, MD, USA) and normalized with β-actin as an internal reference protein.

### 2.11. Statistical Analysis

The data were presented as mean ± SEM. For group comparisons, the one-way analysis of variance (ANOVA) with Tukey’s test was performed using GraphPad Prism 9. Welch’s ANOVA was implemented for data with heterogeneous variances. Statistical significance was defined as *p* < 0.05.

## 3. Results

### 3.1. STA Efficiently Alleviated HUA in Mice

After 4 weeks of intervention, physiological and biochemical parameters were measured in mice. Serum CRE and BUE levels were used to evaluate renal function. As shown in Figure 1C, stachydrine (STA) and benzbromarone (BEN) treatment had no significant effects on the body weight compared with NC and HUA mice. However, the kidney coefficient, serum UA, CRE and BUN levels were significantly increased in HUA mice, whereas STA and BEN reversed these factors except for the kidney coefficient (Figure 1D–G). The results suggested that daily supplementation of 20 and 40 mg/kg STA could effectively alleviate HUA in mice induced by high-nucleoside diet. In cell activity assay, when HK-2 cells were treated with STA (5, 10, 20, 40, 80, 160 and 320 μM) for 48 h, there was no toxic reaction (Figure 1H). Therefore, in subsequent experiments, 20 or 40 μM STA (STA20 or STA40 in this article) was used for HK-2 cells intervention.

### 3.2. STA Mitigated HUA-Induced Renal Inflammation and Pathological Damage

**Figure 2:**
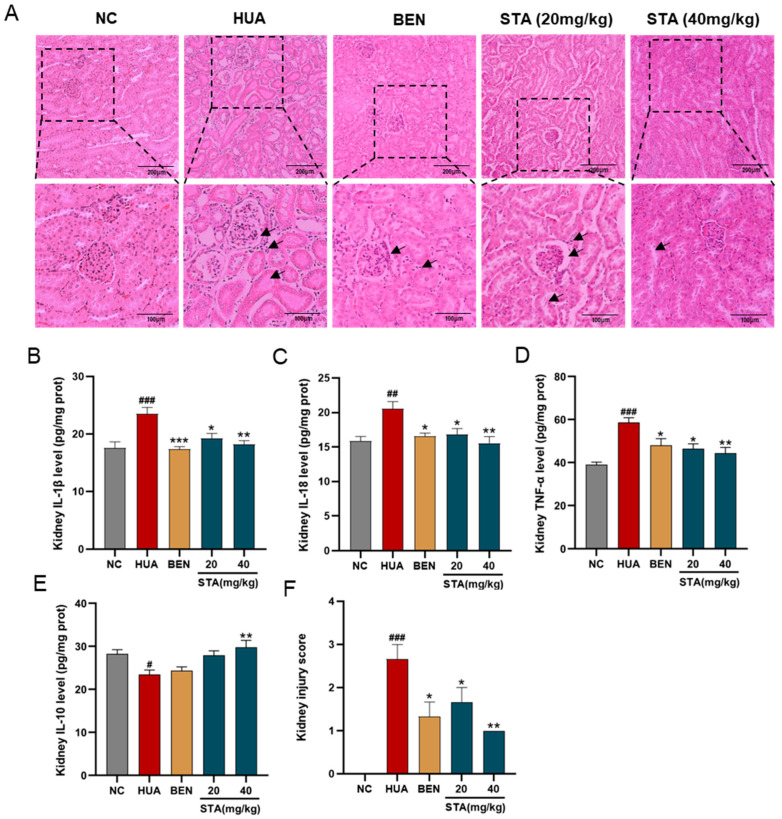
Effects of STA on HUA-induced renal injury. (**A**) Kidney tissues stained with H&E. (**B**–**E**) The levels of IL-1β, IL-18,TNF-α and IL-10. (**F**) Kidney injury score. ^#^ *p* < 0.05, ^##^ *p* < 0.01, ^###^ *p* < 0.001 vs. NC group; * *p* < 0.05, ** *p* < 0.01, *** *p* < 0.001 vs. HUA group.

Histopathological analysis was performed to assess renal injury and inflammation. The renoprotective effects of STA were demonstrated by H&E staining. As illustrated in Figure 2A, HUA mice showed renal injury characterized by glomerular atrophy, tubular dilation and cavitation. Inflammatory markers in kidney tissues were also detected by ELISA, and the results showed that HUA significantly elevated renal levels of proinflammatory cytokines IL-1β, IL-18 and TNF-α, while the anti-inflammatory cytokine IL-10 was decreased (Figure 2B–E), which was consistent with the inflammatory cell infiltration observed in pathological sections. Both BEN and STA reduced IL-1β, IL-18 and TNF-α levels and mitigated renal injury. Particularly, 40 mg/kg STA significantly increased IL-10 levels and improved the kidney injury score (Figure 2F).

### 3.3. STA Enhanced UA Excretion by Upregulating ABCG2

**Figure 3:**
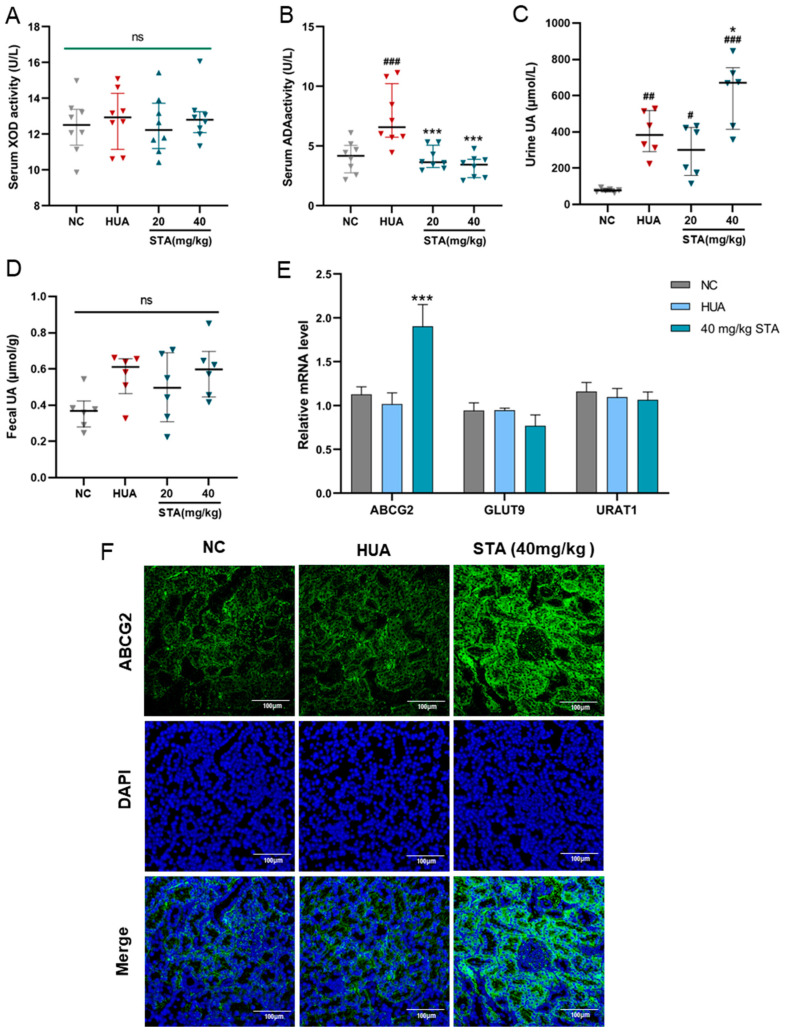
Effects of STA on UA metabolism. (**A**,**B**) Serum XOD and ADA activities in mice. (**C**,**D**) The level of urine and fecal UA. (**E**) Relative mRNA expression of ABCG2, GLUT9 and URAT1 in kidney. (**F**) The protein level of ABCG2 in kidney detected by immunofluorescence. ^#^ *p* < 0.05, ^##^ *p* < 0.01, ^###^ *p* < 0.001 vs. NC group; * *p* < 0.05, *** *p* < 0.001 vs. HUA group.

To further investigate STA’s UA-lowering mechanism, the activities of XOD and ADA were detected, which play pivotal roles in UA biosynthesis. As shown in Figure 3A,B, STA significantly decreased ADA activity but had no inhibitory effect on XOD in serum. However, ADA is not a direct metabolizing enzyme of inosine and guanosine [13]. Next, the focus was on the mice’s UA excretion. Compared with HUA mice, the UA levels in urine were increased in the STA group, while that in feces had no obvious change (Figure 3C,D). It suggested that STA might promote the excretion of UA by the kidneys rather than the intestines. The expression levels of several dominant urate transporters in kidneys were further detected by RT-qPCR. As shown in Figure 3E, the mRNA level of UA secretion protein ABCG2 increased by 1.8-fold compared with the NC group, while GLUT9 and URAT1 did not change significantly. ABCG2 protein expression was also increased in kidney tissues, as detected by immunofluorescence (Figure 3F). The above results indicated that STA might lower UA levels via upregulating renal ABCG2.

### 3.4. STA Mitigated HUA-Induced Renal Mitochondrial Oxidative Stress

**Figure 4:**
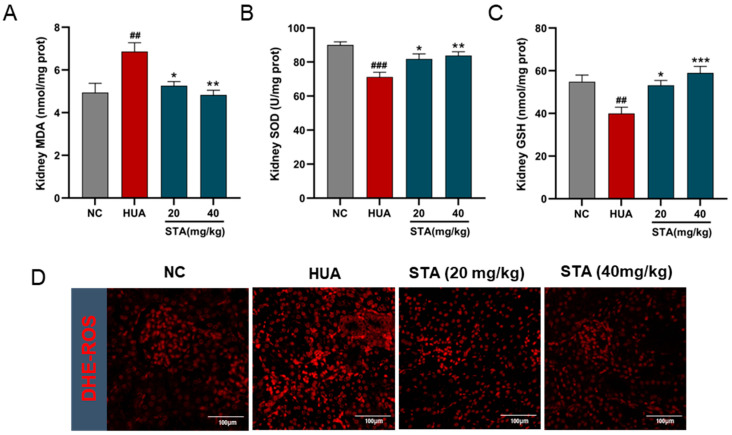
Effects of STA on HUA-induced renal oxidative stress. (**A**–**C**) The levels of MDA, SOD and GSH in kidney tissues. (**D**) ROS in kidney tissues, ^##^ *p* < 0.01, ^###^ *p* < 0.001 vs. NC group; * *p* < 0.05, ** *p* < 0.01, *** *p* < 0.001 vs. HUA group.

Oxidative stress markers in kidney tissues were analyzed. MDA levels indicated ROS-induced damage, while SOD and GSH levels reflected endogenous antioxidant capacity. As shown in Figure 4A–C, MDA levels were increased, while SOD activity and GSH levels were reduced in the HUA mice kidneys, and these indicators were reversed by STA. DHE staining of kidney tissues revealed significant increased ROS in HUA mice, which was effectively decreased after STA supplementation (Figure 4D). This evidence indicated that STA alleviated HUA-induced renal oxidative stress.

**Figure 5:**
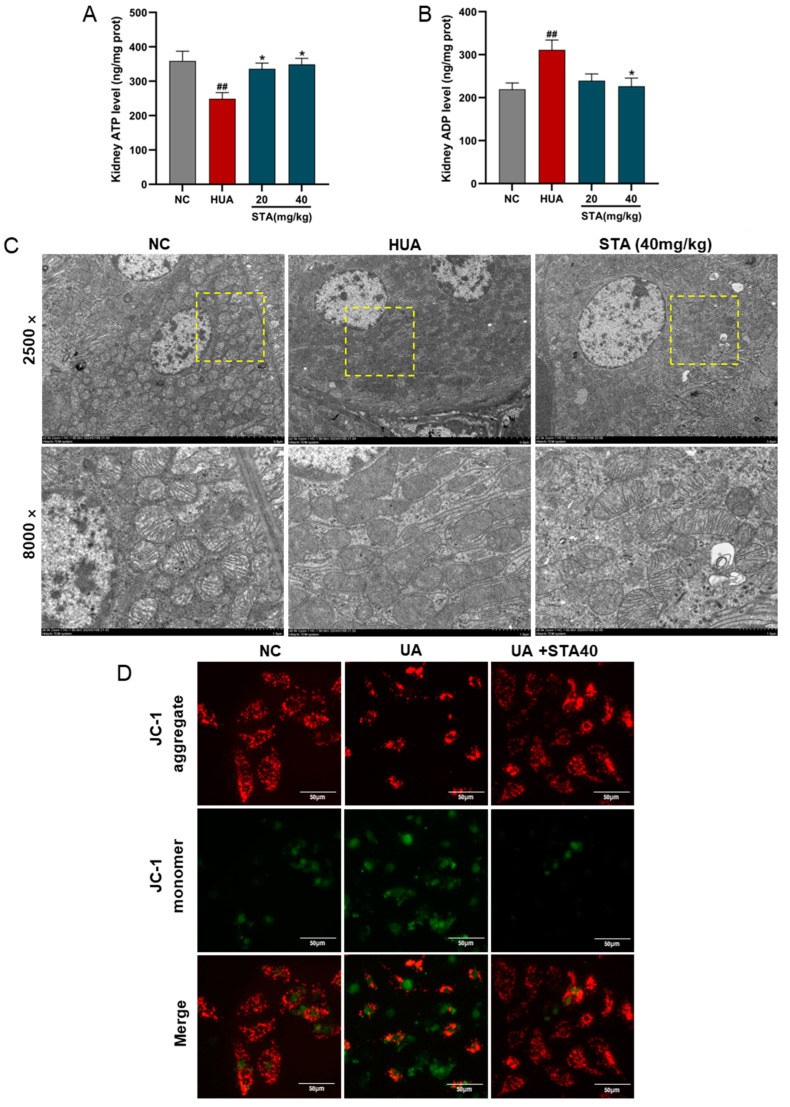
Effects of STA on HUA-induced mitochondrial damage and apoptosis in kidney. (**A**,**B**) The levels of ATP and ADP in kidneys. (**C**) Mitochondrial morphological changes of kidney observed by TEM. The corresponding magnified areas were outlined by dotted lines. (**D**) MMP in HK-2 cells. ^##^ *p* < 0.01, vs. NC group; * *p* < 0.05, vs. HUA group.

Abnormal ATP and ADP levels indicate mitochondrial dysfunction. Kidney tissues analysis revealed ATP depletion and ADP accumulation in the HUA group (Figure 5A,B). STA effectively reversed the contents of ATP and ADP. Further examination of STA’s effect on mitochondrial morphology by TEM revealed that renal mitochondria in the HUA group exhibited swelling, with disorganized, shortened or even absent cristae. Notably, 40 mg/kg STA demonstrated a protective effect on renal mitochondrial structure (Figure 5C). To further demonstrate the effect of STA on mitochondria, the MMP of HK-2 cells was assayed using JC-1. The results showed that high concentrations of UA induced MMP reduction in HK-2 cells, which was significantly attenuated by 40 μM STA (Figure 5D).

**Figure 6:**
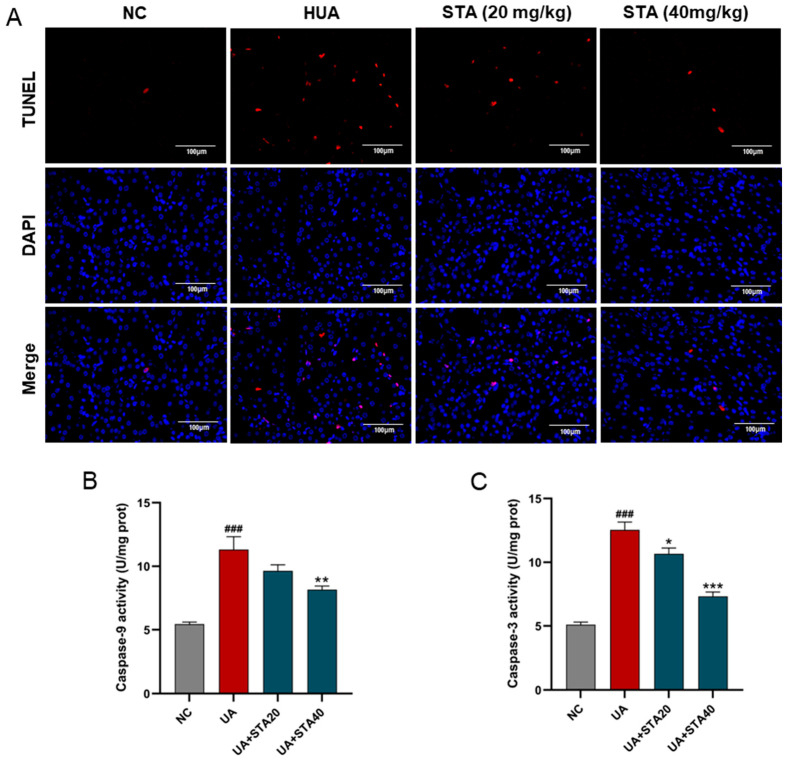
Effects of STA on HUA-induced apoptosis in kidney. (**A**) TUNEL strain of kidney tissues. (**B**,**C**) The activities of caspase-3 and caspase-9 in HK-2 cells. ^###^ *p* < 0.001 vs. NC group; * *p* < 0.05, ** *p* < 0.01, *** *p* < 0.001 vs. UA group.

Mitochondrial damage represents an early and crucial event in caspase-dependent apoptosis, with caspase-9 and caspase-3 as the key markers [10]. Apoptosis in kidney tissues was detected by TUNEL staining. The results revealed that renal cell apoptosis occurred in the HUA group, which was alleviated by STA (Figure 6A). The activities of caspase-9 and caspase-3 were enhanced in HK-2 cells after UA stimulation. Meanwhile, 40 μM STA significantly reduced their activities (Figure 6B,C). These findings collectively indicated that STA preserved renal function by mitigating HUA-induced mitochondrial oxidative stress in the kidney.

### 3.5. STA Alleviated HUA-Induced Oxidative Stress by Regulating the Keap1/Nrf2 Signaling Pathway

**Figure 7:**
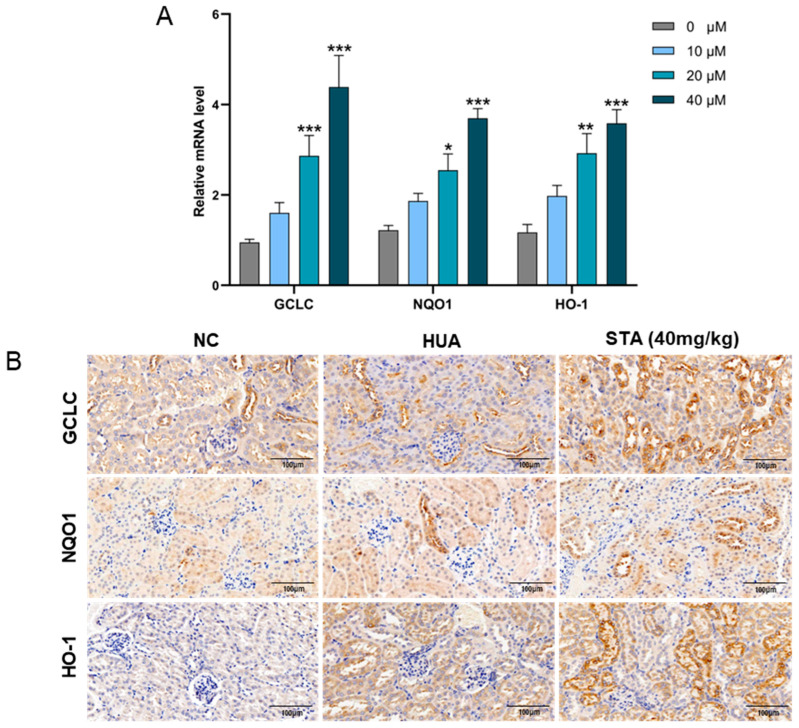
STA affected the expression of antioxidant genes. (**A**) Relative mRNA levels of GCLC, NQO1 and HO-1 in HK-2 cells. (**B**) Immunohistochemical analysis of GCLC, NQO1 and HO-1in kidneys. * *p* < 0.05, ** *p* < 0.01, *** *p* < 0.001 vs. Untreated group.

To evaluate the impact of STA on Nrf2 pathway activation, HK-2 cells were exposed to STA, followed by quantitative assessment of Nrf2-regulated antioxidant genes (GCLC, NQO1 and HO-1) via RT-qPCR. The results showed that STA could enhance their expression (Figure 7A). Immunohistochemical analysis revealed upregulated expression of GCLC, NQO1 and HO-1 proteins in the kidney tissues of the 40 mg/kg STA group (Figure 7B). Their expression levels were also enhanced in HUA mice, which might be caused by HUA-induced oxidative stress.

**Figure 8:**
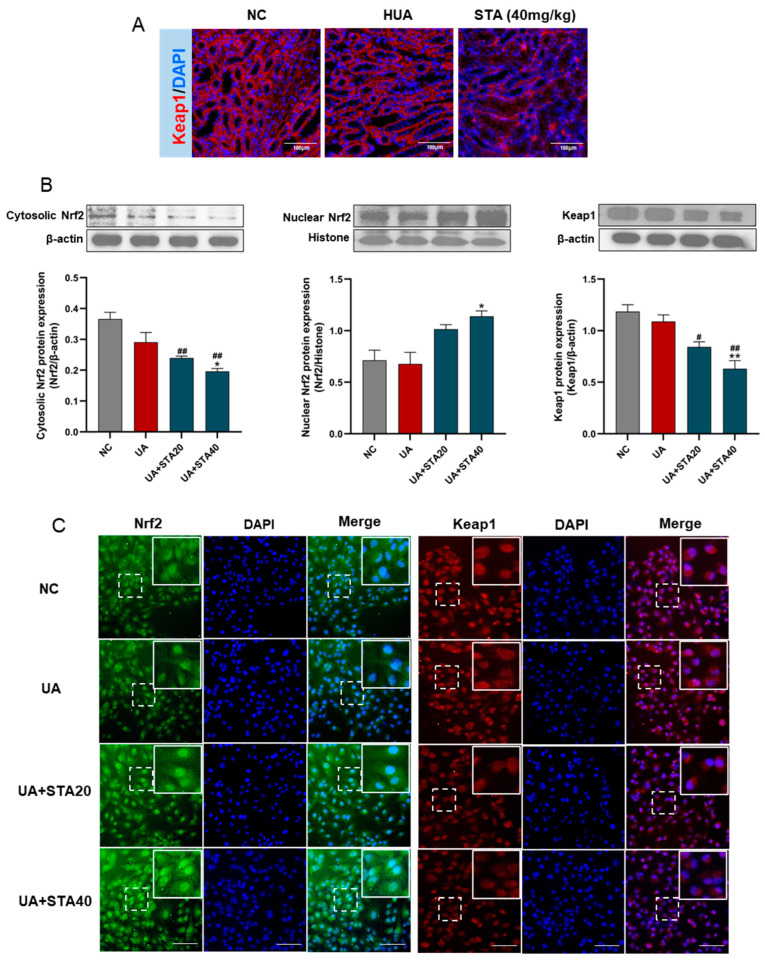
STA regulated Keap1/Nrf2 signaling pathway. (**A**) The protein levels of Keap1 in kidney tissues. (**B**) Western blot and protein quantification analyses for Nrf2 and Keap1 in HK-2 cells. (**C**) Immunofluorescence of Nrf2 and Keap1 in HK-2 cells, and the scale bars represented 100 μm. ^#^ *p* < 0.05, ^##^ *p* < 0.01, vs. NC group; * *p* < 0.05, ** *p* < 0.01, vs. UA group.

To further determine whether STA activates the Nrf2 pathway via Keap1, its protein levels in kidney tissues were detected by immunofluorescence. As shown in Figure 8A, STA treatment reduced Keap1 expression. In addition, the expression changes of Keap1 and Nrf2 were detected by Western blot and immunofluorescence in HK-2 cells treated with STA. The results demonstrated that STA inhibited Keap1 expression and facilitated Nrf2 nuclear translocation (Figure 8B,C).

**Figure 9:**
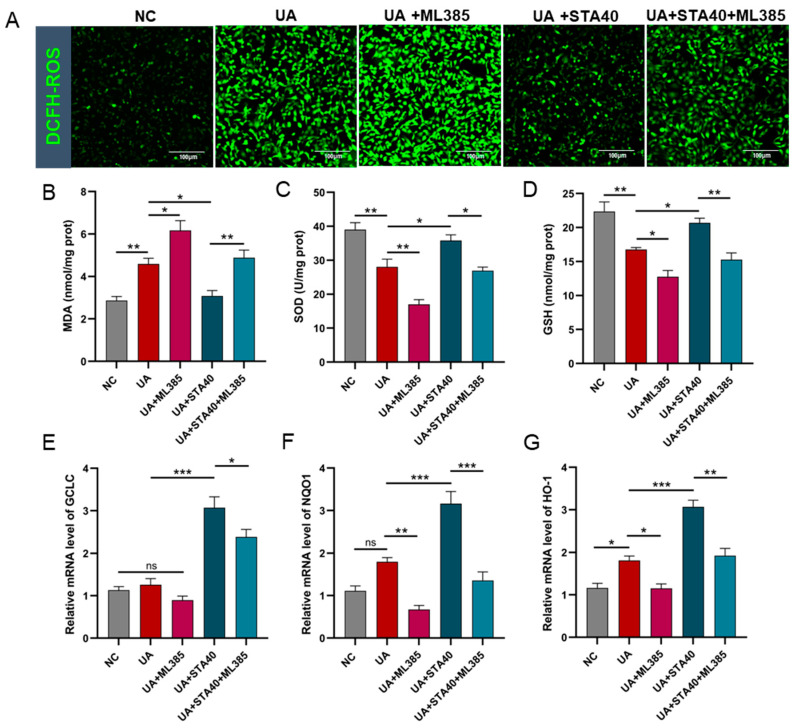
Inhibition of Nrf2 partly abolished STA activity in HK-2 cells. (**A**) ROS levels. (**B**–**D**) The levels of MDA, SOD and GSH. (**E**–**G**) Relative mRNA levels of GCLC, NQO1 and HO-1. * *p* < 0.05, ** *p* < 0.01, *** *p* < 0.001.

For further verification, ML385 was used to specifically inhibit Nrf2, followed by quantification of oxidative stress markers and Nrf2-regulated genes (GCLC, NQO1 and HO-1). The results showed that the inhibition of Nrf2 partly abolished the activity of STA against oxidative stress in HK-2 cells. The ROS in the UA and UA+ML385 groups increased sharply compared with the NC group. 40μM STA effectively suppressed ROS generation, and the ROS levels in the UA+STA40 group were markedly decreased (Figure 9A). Simultaneously, 40 μM STA reduced cellular MDA levels while enhancing SOD and GSH levels; however, these effects were diminished by ML385 co-treatment (Figure 9B–D). Similar patterns were observed in the expression of GCLC, NQO1 and HO-1 (Figure 9E–G). These findings suggested that STA might promote Nrf2 nuclear translocation by inhibiting Keap1 expression and reducing the Keap1/Nrf2 complex in the cytoplasm, thus enhancing the expression of downstream antioxidant genes.

## 4. Discussion

HUA is a pathogenic driver of multiple disease processes, including chronic kidney disease, gout and hypertension. However, the potential adverse reactions of anti-HUA drugs have raised significant clinical concerns [23]. For individuals with HUA, particularly those without overt clinical symptoms such as gout, chemical drug intervention remains controversial. Alkaloids extracted from certain edible plants have been demonstrated to safely lower UA levels. Nuciferine extracted from lotus leaves has been reported to lower UA levels and alleviate renal inflammation in PO-induced HUA mice [24]. Alkaloids extracted from *Peperomia pellucida* showed strong XOD inhibitory activity [25]. Furthermore, Tuberindine A, an alkaloid derived from Truffle, displayed an anti-HUA effect by upregulating urate transporters [26]. This study evaluated the mitigating effects of STA on HUA. STA explicitly reduced serum UA levels in mice via upregulating renal ABCG2. Furthermore, STA demonstrated renoprotective effects. After STA supplementation, renal inflammation and pathological damage in HUA mice were ameliorated. STA mitigated HUA-induced mitochondrial oxidative stress by regulating the Keap1/Nrf2 signaling pathway, thereby attenuating renal cell apoptosis.

Endogenous purine nucleosides are metabolized to UA through the salvage pathway of purine metabolism. In this process, inosine is converted to hypoxanthine by purine nucleoside phosphorylase (PNP), and then catalyzed by XOD to generate xanthine and UA. Guanosine is firstly converted to guanine by PNP, then further metabolized to xanthine, and finally to UA by XOD. Adenosine is converted to inosine by adenosine deaminase (ADA), or to adenine by PNP, and ultimately transformed into hypoxanthine and UA [27]. The reaction catalyzed by PNP is bidirectionally reversible, whereas XOD and ADA act as unidirectional rate-limiting enzymes, making them key regulators of UA synthesis [13]. In vivo experimental results showed that when mice were administered excessive inosine and guanosine, UA accumulated rapidly and was excreted through urine. However, the urine UA content of mice supplemented with 20 mg/kg STA was lower than HUA group, suggesting reduced total UA synthesis. Notably, supplementation with STA did not affect serum XOD activity, but significantly inhibited ADA. Although ADA does not directly catalyze the metabolism of inosine or guanosine, it can convert adenosine to UA. In addition, supplemented inosine can be used to synthesize inosine monophosphate (IMP), which is a precursor of adenosine in the salvage pathway [28]. This may explain an indirect effect that STA likely reduces UA synthesis by suppressing the ADA-dependent endogenous adenosine pathway. Moreover, when UA synthesis was suppressed, the UA excretion in the 40 mg/kg group was still significantly higher than in the HUA and 20 mg/kg groups. These results suggest that enhancing the renal excretion is the primary mechanism by which STA reduces UA.

We postulated that STA primarily reduced UA by enhancing renal efflux. UA homeostasis is maintained by a balance between synthesis and excretion. However, it has been reported that 90% of HUA cases are attributed to UA excretion disorders [29]. ABCG2 is an important urate transporter, highly expressed in kidney and intestinal epithelial cells and mediates the UA excretion [30]. ABCG2 is closely associated with HUA occurrence. Initial studies showed that SNP rs2231142 (Q141K variant) encoding ABCG2 reduced uric acid secretion by 53% compared with the wild type. A population-based study further demonstrated that rs2231142 is strongly associated with gout [31]. Clinical data also demonstrate that Q126X (rs72552713), another nonfunctional ABCG2 variant, significantly increased the risk of HUA [32]. These findings have made ABCG2 an increasingly attractive therapeutic target. Plant-derived natural compounds represent a vast pool for the screening of ABCG2 activators. Huang et al. used Bioaffinity Ultrafiltration Mass Spectrometry to identify fraxin as an activating ligand of ABCG2, which enhances UA transport [33]. Additionally, eurycomanol has been reported to alleviate HUA by upregulating ABCG2 expression in the kidney and intestine [34]. Our study demonstrated that STA promoted UA elimination by upregulating renal ABCG2, providing a potential solution for HUA amelioration, particularly for patients without overt clinical symptoms.

HUA induces ROS by activating NADPH oxidase, and the resulting oxidative stress promotes mitochondrial damage [35]. Mitochondrial damage further increases the production of ROS, which activates NF-κB to trigger the transcription of inflammatory mediators [36]. Mitochondria play a central role in the process of apoptosis. Mitochondrial outer membrane permeabilization (MOMP), triggered by mitochondrial damage, leads to proton gradient disruption and a decreased MMP. Damaged mitochondria release cytochrome c, which subsequently activates the caspase cascade and triggers apoptosis [37]. Our study demonstrated that STA effectively alleviated HUA-induced inflammatory, oxidative stress, mitochondrial damage and cell apoptosis. Further studies found that the Keap1/Nrf2 pathway was a possible antioxidant mechanism of STA for enhancing the local antioxidant response. The Keap1/Nrf2 pathway is one of the most important defense mechanisms for cells to resist oxidative stress [38]. Keap1 is a negative regulator of Nrf2. In normal physiological conditions, Keap1 binds to Nrf2 and is localized in the cytoplasm. When under oxidative stress, the Keap1/Nrf2 complex dissociates, releasing Nrf2 to ectopically enter the nucleus. In the nucleus, Nrf2 binds to ARE, activating the expression of downstream genes [39]. The dissociation of the Keap1/Nrf2 complex plays a pivotal role in initiating this signaling pathway. The Keap1-dependent regulation has attracted researchers’ attention. Small molecule inhibitors targeting Keap1 to activate the pathway have been reported [40,41]. In this study, STA downregulated Keap1 expression, enhanced the translocation of Nrf2 into the nucleus, and subsequently induced the expression of antioxidant enzymes including SOD, GCLC, NQO1 and HO-1. Additionally, endogenous antioxidant GSH levels were elevated in kidney tissue, potentially related to the increased expression of Nrf2-dependent GSH-biosynthesizing enzymes [42].

STA is rapidly absorbed and metabolized following oral administration, demonstrating good bioavailability [43]. Pharmacokinetic studies in a rat model of cold-stagnation and blood-stasis primary dysmenorrhea (CSBS-PD) revealed prolonged retention and enhanced bioavailability of STA in CSBS-PD rats compared to normal controls, suggesting targeted organs distribution [44]. However, detailed bioavailability data for STA remains incomplete. STA is widely distributed in natural foods. Its content in chestnut (*Castanea sativa* Mill.) is 1.1 mg/kg dry weight (DW) [45]. It is present in higher levels in citrus fruits, such as red orange (548 mg/L), yellow orange (486 mg/L), chinotto (455 mg/L) and grapefruit (246 mg/L) [18]. It has been considered a nutritional biomarker of citrus consumption. Excretion kinetics studies indicated that urinary STA levels peaked 2 h after subjects drank orange juice, and 83% was excreted through urine within 14 h [46]. Alfalfa (*Medicago sativa*) and motherwort (*Leonurus*) are also rich in STA, with contents of approximately 0.1% and 0.59–1.72% (DW), respectively [17]. In recent years, dietary patterns and single nutrients have gained attention for their role in mitigating disease risk. Despite its widespread distribution, enrichment of STA in the form of natural foods only is limited. Based on STA’s effects and mechanisms demonstrated in this study, we propose that STA consumption in the form of dietary supplements or fortified foods may be a more feasible approach [47]. Nevertheless, further research is needed to establish safe and effective dosages for humans.

## 5. Conclusions

**Figure 10:**
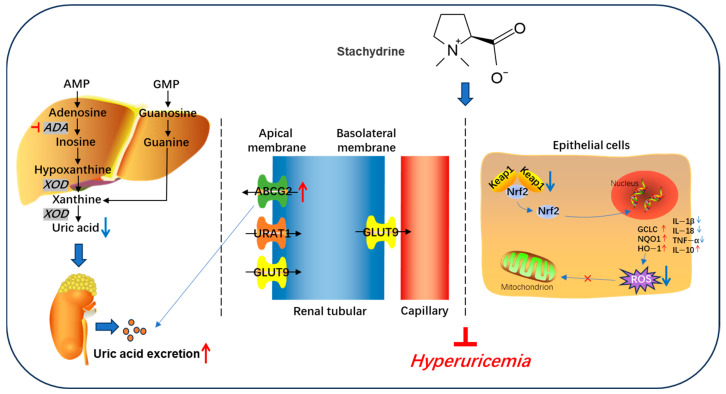
Schematic diagram of stachydrine alleviating hyperuricemia in mice.

In conclusion, STA effectively alleviates HUA by upregulating renal urate transporters ABCG2. Additionally, STA reduces HUA-induced mitochondrial oxidative stress and renal damage. Mechanistically, STA inhibits oxidative stress by regulating the Keap1/Nrf2 signaling pathway (Figure 10). These findings suggest that STA may serve as a potential dietary supplement for managing HUA. Although STA demonstrated significant UA-lowering effects in the mouse model, it requires further validation to establish its applicability as a functional food ingredient in dietary guideline.

## Figures and Tables

**Table 1:** The specific primers sequences (5′–3′) used in RT-qPCR.

Gene	Species	Forward Sequence	Reverse Sequence
*ABCG2*	Mouse	GAACTCCAGAGCCGTTAGGAC	CAGAATAGCATTAAGGCCAGGTT
*GLUT9*	Mouse	TTGCTTTAGCTTCCCTGATGTG	GAGAGGTTGTACCCGTAGAGG
*URAT1*	Mouse	TGGGTTTACGACCACAGCAC	CTTCTGCGCCCAAACCTATCT
*GCLC*	Human	TACGGAGGAACAATGTCCGA	CAGTGTGAACCCAGGACAGC
*NQO1*	Human	AAGAAGAAAGGATGGGAGGTGG	GAACAGACTCGGCAGGATACTG
*HO-1*	Human	GCCAGCAACAAAGTGCAAGA	TAAGGACCCATCGGAGAAGC
*β-actin*	Mouse	GAGACCTTCAACACCCCAGC	ATGTCACGCACGATTTCCC
*β-actin*	Human	TTGGCAATGAGCGGTTCC	AGACAGCACTGTGTTGGC

## Data Availability

The original contributions presented in the study are included in the article, further inquiries can be directed to the corresponding authors.

## References

[1] Jiang L.L., Gong X., Ji M.Y., Wang C.C., Wang J.H., Li M.H. (2020). Bioactive compounds from plant-based functional foods: A promising choice for the prevention and management of hyperuricemia. Foods.

[2] Yang L., Wang B., Ma L., Fu P. (2022). Traditional Chinese herbs and natural products in hyperuricemia-induced chronic kidney disease. Front. Pharmacol..

[3] Barman Z., Hasan M., Miah R., Mou A.D., Hafsa J.M., Trisha A.D., Mahmud F., Ali N. (2023). Association between hyperuricemia and chronic kidney disease: A cross-sectional study in Bangladeshi adults. BMC Endocr. Disord..

[4] Riaz M., Al Kury L.T., Atzaz N., Alattar A., Alshaman R., Shah F.A., Li S. (2023). Carvacrol alleviates hyperuricemia-induced oxidative stress and inflammation by modulating the NLRP3/NF-κB pathway. Drug Des. Dev. Ther..

[5] Yang B., Xin M., Liang S., Xu X., Cai T., Dong L., Wang C., Wang M., Cui Y., Song X. (2022). New insight into the management of renal excretion and hyperuricemia: Potential therapeutic strategies with natural bioactive compounds. Front. Pharmacol..

[6] Su H.Y., Yang C., Liang D., Liu H.F. (2020). Research advances in the mechanisms of hyperuricemia-induced renal injury. Biomed. Re. Int..

[7] Liu Y.L., Gong S.T., Li K.J., Wu G., Zheng X.H., Zheng J.N., Lu X.W., Zhang L.Y., Li J.C., Sun Z.R. (2022). Coptisine protects against hyperuricemic nephropathy through alleviating inflammation, oxidative stress and mitochondrial apoptosis via PI3K/Akt signaling pathway. Biomed. Pharmacother..

[8] Li X.Y., Zhang W., Cao Q.T., Wang Z.Y., Zhao M.Y., Xu L.Y., Zhuang Q. (2020). Mitochondrial dysfunction in fibrotic diseases. Cell Death Discov..

[9] Cristóbal-García M., García-Arroyo F.E., Tapia E., Osorio H., Arellano-Buendía A.S., Madero M., Rodríguez-Iturbe B., Pedraza-Chaverrí J., Correa F., Zazueta C. (2015). Renal oxidative stress induced by long-term hyperuricemia alters mitochondrial function and maintains systemic hypertension. Oxid. Med. Cell. Longev..

[10] Zhuang C., Ni S., Yang Z.C., Liu R.P. (2020). Oxidative stress induces chondrocyte apoptosis through caspase-dependent and caspase-independent mitochondrial pathways and the antioxidant mechanism of angelica sinensis polysaccharide. Oxid. Med. Cell Longev..

[11] Sun H.J., Ding S., Guan D.X., Ma L.Q. (2022). Nrf2/Keap1 pathway in countering arsenic-induced oxidative stress in mice after chronic exposure at environmentally-relevant concentrations. Chemosphere.

[12] Liu N., Xu H., Sun Q., Yu X., Chen W., Wei H., Jiang J., Xu Y.Z., Lu W. (2021). The role of oxidative stress in hyperuricemia and xanthine oxidoreductase (XOR) inhibitors. Oxid. Med. Cell. Longev..

[13] Lin S.M., Meng J., Li F., Yu H.F., Lin D.M., Lin S.Q., Li M., Zhou H., Yang B.X. (2022). Ganoderma lucidum polysaccharide peptide alleviates hyperuricemia by regulating adenosine deaminase and urate transporters. Food Funct..

[14] Adomako E.A., Moe O.W. (2023). Uric acid transport, transporters, and their pharmacological targeting. Acta Physiol..

[15] Rey A., Batteux B., Laville S.M., Marienne J., Masmoudi K., Gras-Champel V., Liabeuf S. (2019). Acute kidney injury associated with febuxostat and allopurinol: A post-marketing study. Arthritis Res. Ther..

[16] Ye X., Wu J., Tang K., Li W., Xiong C., Zhuo L. (2019). Benzbromarone as a possible cause of acute kidney injury in patients with urolithiasis: Two case reports. Medicine.

[17] He Z., Li P., Liu P., Xu P. (2024). Exploring stachydrine: From natural occurrence to biological activities and metabolic pathways. Front. Plant Sci..

[18] Ahmed S.A., Borah J.C., Manna P. (2024). Stachydrine, a pyrrole alkaloid with promising therapeutic potential against metabolic syndrome and associated organ dysfunction. RSC Med. Chem..

[19] Chen H.H., Zhao P., Zhao W.X., Tian J., Guo W., Xu M., Zhang C., Lu R. (2017). Stachydrine ameliorates pressure overload-induced diastolic heart failure by suppressing myocardial fibrosis. Am. J. Transl. Res..

[20] Liao L., Tang Y., Li B., Tang J., Xu H., Zhao K., Zhang X. (2023). Stachydrine, a potential drug for the treatment of cardiovascular system and central nervous system diseases. Biomed. Pharmacother..

[21] Zhang C., Lu Y., Tong Q.Q., Zhang L., Guan Y.F., Wang S.J., Xing Z.H. (2013). Effect of stachydrine on endoplasmic reticulum stress-induced apoptosis in rat kidney after unilateral ureteral obstruction. J. Asian Nat. Prod. Res..

[22] Sun L., Liu Q., Zhang Y., Xue M., Yan H., Qiu X., Tian Y.J., Zhang H.Q., Liang H. (2023). Fucoidan from *Saccharina japonica* alleviates hyperuricemia-induced renal fibrosis through inhibiting the JAK2/STAT3 signaling pathway. J. Agric. Food Chem..

[23] Mehmood A., Zhao L., Wang C., Nadeem M., Raza A., Ali N., Shah A.A. (2019). Management of hyperuricemia through dietary polyphenols as a natural medicament: A comprehensive review. Crit. Rev. Food Sci..

[24] Wang M.X., Liu Y.L., Yang Y., Zhang D.M., Kong L.D. (2015). Nuciferine restores potassium oxonate-induced hyperuricemia and kidney inflammation in mice. Eur. J. Pharmacol..

[25] Fachriyah E., Ghifari M.A., Anam K. (2018). Isolation, Identification, and Xanthine oxidase inhibition activity of alkaloid compound from *Peperomia pellucida*. IOP Mater. Sci. Eng..

[26] Zhao L.Q., Zhao Y.L., He Y.J., Yang X.W., Luo X.D. (2022). Tuberindine a, a truffle alkaloid with an unprecedented skeleton exhibiting anti-hyperuricemic bioactivity. Org. Lett..

[27] Feng S., Wu S., Xie F., Yang C.S., Shao P. (2022). Natural compounds lower uric acid levels and hyperuricemia: Molecular mechanisms and prospective. Trends Food Sci. Tech..

[28] Mileti L.N., Baleja J.D. (2025). The role of purine metabolism and uric acid in postnatal neurologic development. Molecules.

[29] Han Y., Liu W.L., Li K.X., Zhang M.Z., Liu X.Q., Li L., Guo Z., Li H. (2024). Investigating the role of food-derived peptides in hyperuricemia: From mechanisms of action to structural effects. Foods.

[30] Takada T., Miyata H., Toyoda Y., Nakayama A., Ichida K., Matsuo H. (2024). Regulation of urate homeostasis by membrane transporters. Gout Urate Cryst. Depos. Dis..

[31] Woodward O.M., Köttgen A., Coresh J., Boerwinkle E., Guggino W.B., Köttgen M. (2009). Identification of a urate transporter, ABCG2, with a common functional polymorphism causing gout. Proc. Natl. Acad. Sci. USA.

[32] Matsuo H., Takada T., Nakayama A., Shimizu T., Sakiyama M., Shimizu S., Seiko S., Chiba T., Nakashima H., Nakamura T. (2014). ABCG2 dysfunction increases the risk of renal overload hyperuricemia. Nucleos. Nucleot. Nucl..

[33] Huang X., Dong W., Luo X., Xu L., Wang Y. (2023). Target screen of anti-hyperuricemia compounds from Cortex Fraxini in vivo based on ABCG2 and bioaffinity ultrafiltration mass spectrometry. Molecules.

[34] Bao R.X., Chen Q., Li Z., Wang D., Wu Y.Z., Liu M.Y., Zhang Y., Wang T. (2022). Eurycomanol alleviates hyperuricemia by promoting uric acid excretion and reducing purine synthesis. Phytomedicine.

[35] Copur S., Demiray A., Kanbay M. (2022). Uric acid in metabolic syndrome: Does uric acid have a definitive role?. Eur. J. Intern. Med..

[36] Chen A., Huang H., Fang S., Hang Q. (2024). ROS: A “booster” for chronic inflammation and tumor metastasis. BBA-Rev. Cancer.

[37] Bock F.J., Tait S.W. (2020). Mitochondria as multifaceted regulators of cell death. Nat. Rev. Mol. Cell Biol..

[38] Tu W., Wang H., Li S., Liu Q., Sha H. (2019). The anti-inflammatory and anti-oxidant mechanisms of the Keap1/Nrf2/ARE signaling pathway in chronic diseases. Aging Dis..

[39] Jayasuriya R., Dhamodharan U., Ali D., Ganesan K., Xu B., Ramkumar K.M. (2021). Targeting Nrf2/Keap1 signaling pathway by bioactive natural agents: Possible therapeutic strategy to combat liver disease. Phytomedicine.

[40] Hamdy S., Elshopakey G.E., Risha E.F., Rezk S., Ateya A.I., Abdelhamid F.M. (2024). Curcumin mitigates gentamicin induced-renal and cardiac toxicity via modulation of Keap1/Nrf2, NF-κB/iNOS and Bcl-2/BAX pathways. Food Chem. Toxicol..

[41] Feng J.Z., Ji K.B., Pan Y.J., Huang P.P., He T., Xing Y. (2024). Resveratrol ameliorates retinal ischemia-reperfusion injury by modulating the Nlrp3 inflammasome and Keap1/Nrf2/Ho-1 signaling pathway. Mol. Neurobiol..

[42] Piao M.J., Fernando P.M.D.J., Kang K.A., Fernando P.D.S.M., Herath H.M.U.L., Kim Y.R., Hyun J.W. (2024). Rosmarinic acid inhibits ultraviolet b-mediated oxidative damage via the AKT/ERK-NRF2-GSH pathway in vitro and in vivo. Biomol. Ther..

[43] Sun Y.F., Xia X.X., Yuan G.J., Zhang T.K., Deng B.B., Feng X.Y., Wang Q.X. (2023). Stachydrine, a bioactive equilibrist for synephrine, identified from four citrus Chinese herbs. Molecules.

[44] Wen Y.Q., Gong L.Y., Wang L., Zhao N., Sun Q., Kamara M.O., Ma H.Y., Meng F.H. (2019). Comparative pharmacokinetics study of leonurine and stachydrine in normal rats and rats with cold-stagnation and blood-stasis primary dysmenorrhoea after the administration of Leonurus japonicus houtt electuary. J. Sep. Sci..

[45] Servillo L., Giovane A., Casale R., Balestrieri M.L., Cautela D., Paolucci M., Siano F., Volpe M.G., Castaldo D. (2016). Betaines and related ammonium compounds in chestnut (*Castanea sativa* Mill.). Food Chem..

[46] Heinzmann S.S., Brown I.J., Chan Q., Bictash M., Dumas M.E., Kochhar S., Stamler J., Holmes E., Elliott P., Nicholson J.K. (2010). Metabolic profiling strategy for discovery of nutritional biomarkers: Proline betaine as a marker of citrus consumption. Am. J. Clin. Nutr..

[47] Domínguez Díaz L., Fernández-Ruiz V., Cámara M. (2020). The frontier between nutrition and pharma: The international regulatory framework of functional foods, food supplements and nutraceuticals. Crit. Rev. Food Sci..

